# circRNA/miRNA Networks Regulate KLF4 in Tumor Development

**DOI:** 10.3390/ncrna11040056

**Published:** 2025-07-29

**Authors:** Raffaele Frazzi, Enrico Farnetti, Davide Nicoli

**Affiliations:** Molecular Pathology Laboratory, Azienda Unità Sanitaria Locale—IRCCS di Reggio Emilia, 42123 Reggio Emilia, Italy

**Keywords:** *KLF4*, epigenetic regulation, circRNAs

## Abstract

**Background/Objectives**: Krüppel-like factor 4 (*KLF4*) emerged as an epigenetically regulated gene in a variety of settings, including cell reprogramming and malignant cell proliferation. The aim of the present manuscript is to explore the relationship described in recent years between circular RNAs, miRNAs, and *KLF4*. These have been shown to be involved in cancers having diverse histological origins, including some of the most prevalent and deadly tumors for the human population. Expression and protein levels of this transcription factor correlate with invasiveness and prognosis in a context- and tissue-specific fashion. **Methods**: The literature was obtained through two main PubMed queries. The first is “miRNA and KLF4 and cancer” and is limited to the last 5 years. The second is “circRNA and KLF4”, which yielded publications between 2013 and 2024. The oncological publications were selected. **Results**: A number of circRNA/miRNA axes that regulate the downstream transcription factor *KLF4* emerged in the last few years. circRNAs act as sponges for miRNAs and synergize with *KLF4*, which can function as either a tumor promoter or suppressor in different tumors. **Conclusions**: The axes represented by circRNA/miRNA/*KLF4* emerged as a new layer of epigenetic regulation. These RNA-based modulators explain the complex regulation of this transcription factor and open the way to new therapeutic targeting possibilities.

## 1. Introduction

Krüppel-like factor 4 (*KLF4*) is a zinc-finger transcription factor widely involved in cell reprogramming towards pluripotency and associated with cancers of different histological origin [1,2]. The last decades highlighted a surge in research and interest concerning non-coding RNAs (ncRNAs). ncRNAs represent the principal product of eukaryotic transcription and include different classes of molecules (aside from ribosomal and transfer RNA) playing essential regulatory functions for gene expression [3]. Some ncRNAs are reportedly involved in the modulation of the expression and function of the transcription factor *KLF4* [4].

Despite the fact that a number of studies demonstrate a role as tumor suppressor [5,6,7], some recent data also highlight a role as tumor promoter, underlying the need for a specific and context-dependent evaluation [4,8,9,10].

The ncRNAs that emerged in the present manuscript belong to the classes of microRNAs (miRNAs) [8] and circular RNAs (circRNAs) [11]. The great heterogeneity of regulatory ncRNAs interacting with *KLF4*, together with the limited knowledge of the mechanisms of action of circRNAs, make this topic particularly challenging.

In the present manuscript the most recent data available on miRNAs are described first, then the ones concerning the circRNAs focused on cancer. A number of circRNA/miRNA axes that regulate the downstream *KLF4* mRNA emerged as a layer of post-transcriptional regulation. A network of biological processes built on circRNAs and *KLF4* is then produced through the use of CircAtlas and Cytoscape software. Finally, some remarkable examples of stage-dependent epigenetic regulation of *KLF4* are reported, with the specific aim of explaining the need for a context-dependent assessment of *KLF4* levels/involvement. The issue of the context-dependent functions in tumors has been pointed out by the recent literature and acknowledged by the scientific community.

The main circRNA/miRNA axes aiming at regulating *KLF4* described by the recent literature are reported and exploited in the present manuscript with the aim of offering hints for new therapeutic opportunities. This epigenetic regulation emerges as a potentially relevant mechanism of modulation in the chemoresistance, stemness, and progression of the cancer cells. *KLF4* expression is eventually modulated through the interaction between these classes of ncRNAs, opening the way to novel and targeted therapeutic antitumoral approaches.

## 2. KLF4 Is a Target for miRNAs

**Figure 1:**
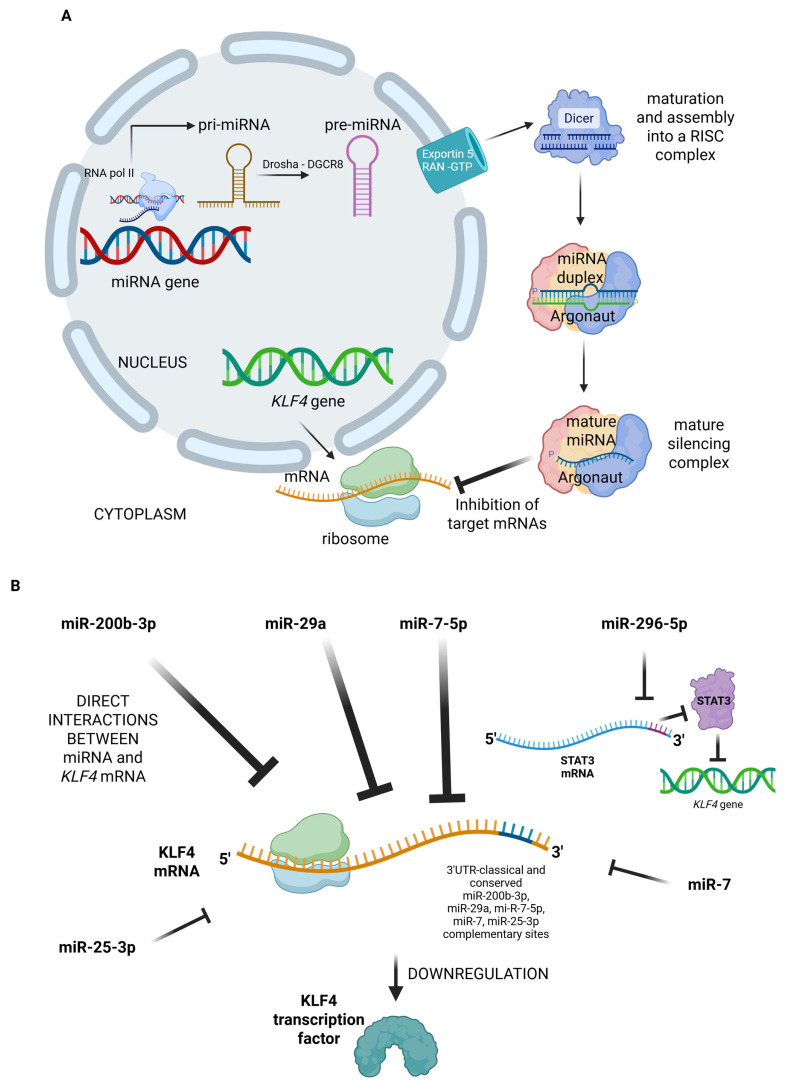
(**A**) Representation of the inhibition of *KLF4* messenger RNA (mRNA) by the complex constituted by micro RNA (miRNA) and by the proteins of the ribonucleic complex (RISC complex). *KLF4* gene is schematically represented and the mRNA originated in the nucleus is transferred to the cytoplasm where the imperfect complementary pairing between the miRNAs and the conserved sites at 3′-UTR of the mRNA happens, leading to translational repression. (**B**) Direct (miR-200b-3p, miR-29a, miR-7-5p, miR-7, miR-25-3p) and indirect (miR-296-5p) inhibitory activity on *KLF4* mRNA. Images were prepared with bioRender (www.biorender.com).

Several authors unveiled the links existing between families of miRNAs and the gene regulation of *KLF4*. As is known, the 3′-untranslated region (3′-UTR) of the transcribed messenger RNA (mRNA) is targeted by the RNA-induced silencing complex (RISC), a complex containing the specific miRNA. Base complementarity between sequences in the target 3′-UTR and miRNAs leads to pairing and eventual downregulation of the specific target transcripts [8]. This mechanism therefore belongs to post-transcriptional regulation (Figure 1A).

The database Targetscan is a tool designed by a group from Massachusetts Institute of Technology led by Bartel D.P. Targetscan 8.0 contributes to the identification of the targets of the different families of miRNAs since it works as a search engine. It provides the families of miRNAs known to interact with 3′-UTR of a given target mRNA, yielding detailed information on their exact pairing position and conserved consensus sequences [12,13,14]. This information thus helps in identifying which miRNAs can inhibit *KLF4* and, indirectly, its targets (Figure 1B). *KLK4* emerges as a target for miR-200b-3p on Targetscan 8.0 [12]. The inhibition of *KLF4*-mediated autophagy in cholangiocarcinoma (CCA) cells can be mediated by miR-200b-3p and leads to an increased sensitivity to 5-FU treatment [8]. Interestingly, cholangiocarcinoma’s first line of therapy is represented by 5-FU, and this is consistent with a role played by the axis miR-200b-3p-*KLF4* in tumor chemoresistance. Investigation of CCA tissue sections also shows that *KLF4* expression and protein levels are higher in CCA samples compared to matched nontumor tissues. These data collectively support the hypothesis that *KLF4* promotes CCA tumorigenesis and 5-FU resistance [8].

miR-296-5p is another regulator of *KLF4* through its activity on STAT3, even though the data have been reported for nasopharyngeal carcinoma cell lines and mouse models and have yet to be confirmed on a larger scale [15]. STAT3 targets *KLF4* and their expression is positively correlated. *KLF4* is thus indirectly modulated by miR-296-5p and plays an oncogenic role in this setting [15].

The modulation mediated by miRNAs is also reported in highly prevalent and/or incurable human cancers like triple-negative breast cancer (TNBC). Cancer stem cells (CSCs) are known to be tumor-initiating cells and critical players in recurrence, therapy resistance, and metastasis [16]. miR-29a has emerged as a target for estrogen receptor alpha in breast cancer (BC) cells [17]. The overexpression of miR-29a led to an arrest of tumor initiation and a decrease in CSCs in TNBC by directly targeting *KLF4* [16]. This inhibition is concordant with the requirement of *KLF4* for the reprogramming towards stemness, as part of the OSKM (OCT3/4, SOX2, KLF4, and c-MYC) transcription factors (TFs) [2].

The demonstration that miR-7-5p directly binds to 3′-UTR and inhibits the *KLF4* transcript has been published recently [18]. *KLF4* expression promotes radioresistance of colorectal cancer (CRC) cell lines, and its tumor-promoting role seems to be also confirmed in patient-derived xenograft (PDX) models of CRC. Overexpression of miR-7-5p leads to the recovery of radiosensitivity and to a decrease in the proliferation potential of the PDX, supporting a potential therapeutic use of this miRNA [18]. Another oncosuppressive activity of miR-7-5p in CRC was described by Dong M. et al., who had already demonstrated how *KLF4* was a direct target of this miRNA while the same network was also shown in childhood nephroblastoma [19,20]. miR-7-5p expression levels were lower in the CRC tissues compared to adjacent, nontumor counterparts, and the overall survival of CRC patients was lower in the ones with lower miR-7-5p expression by quantitative real-time PCR [19]. Targetscan analysis eventually unveiled that miR-7-5p directly targets *KLF4* by means of 3′-UTR recognition and binding (a mechanism already reported above [12]) (Figure 1B). These layers of post-transcriptional downregulation thus point to a tumor-promoting and radioresistance-promoting role of *KLF4* in CRC, at variance with previously published data on this neoplasy. These contradictory studies point towards a tightly regulated expression of *KLF4*, possibly reflecting specific functions during different phases of CRC. Furthermore, miR-7-5p was previously demonstrated to perform tumor-suppressive functions in several malignancies like breast, lung, and hepatocellular carcinomas, thus supporting the recent studies on CRC [21,22,23].

The tumor-suppressive properties of miRNAs have been further investigated by the researchers in order to develop efficient methods to initiate reprogramming towards pluripotency. The direct transfection and expression of miR-200c, miR-302s, and miR-369s may lead to the re-expression of pluripotency factors through the demethylation processes of the target transcription factors [24,25]. The interplay between miRNAs and specific TFs is recognized as pivotal in maintaining the self-renewal and differentiation potential of CSCs [26]. miR-7, for instance, suppresses *KLF4* in breast CSCs, whereas miR-25-3p (a metastasis-promoting miRNA) targets *KLF4* and eventually vascular permeability and angiogenesis in CRC [27,28].

The topic of whether *KLF4* acts as a tumor suppressor or a tumor promoter specifically in CRC is still controversial, since there is evidence supporting either one or the other function. An intriguing hypothesis is that *KLF4* can be switched off during different stages of the disease by means of one or more of the several known epigenetic layers of regulation.

These studies, collectively, suggest a great potential for the therapeutic targeting of *KLF4* in CRC by means of circRNA/miRNA networks. The advent of anti-miRNA, miRNA-mimics, and antisense oligonucleotide-based therapies, for instance, represents an expanding opportunity for targeted therapy since the recognition sequence can be adapted to the target [29].

## 3. KLF4 Is a Target for circRNAs

Circular RNAs (circRNAs) represent one of the most recent noncoding-RNA classes being characterized. There are far more than 1,000,000 different circRNAs annotated thus far, more than 768,000 of which have been characterized in humans. A variety of regulatory functions have been attributed to these novel molecules [30]. circRNAs originate by alternative splicing of a transcript, through a process defined as “backsplicing”. circRNAs contain the fragments generated by alternative splicing [31]. During backsplicing a downstream splice donor site is linked to an upstream splice acceptor site by a covalent bond (5′-3′), and therefore circRNAs are more stable than their host gene-derived linear mRNAs [32]. The proximity of these two splicing sites may lead to backsplicing through the canonical splicing machinery [11]. circRNAs can be sequenced upon enrichment through RNase R treatment, do not contain poly(A) tails, and are of smaller size than the linear, isogenic mRNAs [11]. Although circRNAs are mainly localized in the cytoplasm, where they exert a regulatory activity on target transcripts, studies also demonstrate their presence in the nucleus [31]. The great stability of circRNAs (due to their biochemical structure) together with their tissue specificity makes them an extraordinary tool as novel biomarkers. circRNAs retain the exons present in the linear transcripts and may also encompass exons that are not normally transcribed and therefore absent in the linear counterparts [31]. Notably, methylation of gene bodies linked to DNMT3B directly affects the level of circRNAs in a way that is independent of the expression levels of the corresponding linear host genes [33].

Diverse roles emerged for circRNAs in gene regulation, development, and carcinogenesis. One of the main and first-to-be-discovered functions of circRNAs is to sponge miRNAs. This is achieved because the products of alternative splicing (backsplicing) contain the sequences complementary to the specific miRNAs. miRNAs thus bind to the circRNA product and not to the linear, coding mRNA yielding indirectly to the upregulation of downstream targets. Therefore circRNAs affect *KLF4* expression indirectly, often by acting on specific miR-*KLF4* axes (Table 1). For instance, in colorectal cancer (CRC) circRNAs affect the axis miR-29a-3p-*KLF4* [34].

The resource circAtlas 3.0 provides a curated database and a variety of tools for circRNA identification and analysis [30]. The queries yield the circAtlas ID and the coordinates in the hg38 assembly, together with information like length, nucleotide sequence, tissue distribution, and secondary structure [30]. The database also displays the expression levels of circRNAs and related genes.

ciRS-7 (Circular RNA Sponge For MiR-7, also called Cerebellar Degeneration Related 1 antisense, CDR1as; the current name of CDR1as according to the HUGO gene nomenclature committee is long intergenic non-protein coding RNA 632, LINC00632) has been described as a tumor promoter in CRC and esophageal squamous cell carcinoma and affects *KLF4* expression in hepatocellular carcinoma (HCC) [31,35,36]. This last function is achieved by sponging miR-7-5p, a miRNA that downregulates *KLF4*, eventually promoting cell proliferation and stemness (Figure 1B) [4]. It is estimated that CDR1as contains more than 70 conserved binding sites for miR-7, representing a competing endogenous RNA for this miRNA [37].

CDR1as (current name: long intergenic non-protein coding RNA 632, LINC00632) modulates several miRNA-regulated networks while miR-7 interaction is the best documented thus far [35,38,39]. miR-7-5p sponges directly *KLF4* in hepatoblastoma while miR-7 in esophageal carcinoma [35,40]. The recently discovered circRNA circ_0015756 is overexpressed in hepatoblastoma and HCC [41,42]. Its target is, again, the oncosuppressive miR-7 whose expression is inversely correlated to circ_0015756 in both HCC tissues and cell lines. *KLF4* is kept downregulated by miR-7 that eventually inhibits HCC progression [43].

CircPRMT5 (circRNA derived from Protein Arginine Methyltransferase 5 gene) affects the axis miR-7-5p/*KLF4* in the rare pediatric tumor nephroblastoma. Specifically, inverse correlations between circ-PRMT5-miR-7-5p and between miR-7-5p-*KLF4* have been observed (total of *n* = 45 Wilms’ tumor samples) [20].

CircLECRC (circRNA low expressed in CRC) is a circRNA derived from the oncogene Yes1 Associated Transcriptional Regulator (*YAP1*) [44]. It works by sponging miR-135b-5p and relieving its inhibition on the target *KLF4* in CRC. circLECRC and *KLF4* work together as tumor suppressors by inhibiting *YAP1* hyperactivation and eventually its downstream pathway (EGFR, MYC, BIRC5, and CTGF) [44]. In a previous work conducted on a total of 91 CRC specimens and their matched adjacent nontumor tissues, 4735 circRNAs were obtained by filtered RNA deep sequencing. Among these, 15 competing endogenous RNAs specific to *KLF4* were selected [45]. *KLF4* is known to suppress CRC by modulating Notch and Wnt/β-catenin pathways. Ge J. et al. therefore selected 15 circRNAs targeting *KLF4* and validated one of them (circ_0142527). Both circ_0142527 and *KLF4* were lower in tumor tissues [45].

Non-small cell lung cancer (NSCLC) biopsies display a significantly higher level of circUBAP2 (circRNA derived from Ubiquitin Associated Protein 2) compared to matched normal tissue samples, as reported by Zheng G. et al. [46]. circUBAP2 acts by targeting miR-3182 that keeps *KLF4* downregulated. The specificity of miR-3182 for *KLF4* was predicted in silico and verified on NSCLC cell lines, and *KLF4* expression is inversely and consistently correlated to miR-3182. Overexpression of circUBAP2 promotes survival and proliferation of NSCLC cells by targeting the miR-3182-*KLF4* axis [46].

circRNAs have also been shown to modulate *KLF4* in BC. circEHMT1 (circRNA derived from histone-lysine methyltransferase 1) was downregulated in breast cancer specimens compared to their normal counterpart and served as a suppressor of migration and invasion when overexpressed in BC cell lines [47]. The target miRNA is miR-1233-3p and is upregulated in BC tissues compared to their normal ones; this is consistent with the sponging activity exerted by circEHMT1 in the nonmalignant cells. *KLF4* is one of the five known targets of miR-1233-3p, and it emerged that it was responsible for metalloproteinase-2 (MMP2) inhibition in the axis circEHMT1/miR-1233-3p/*KLF4* [47].

circPLEKHM3 (circRNA derived from Pleckstrin Homology Domain Containing M3) emerged as one of the most downregulated circRNAs in ovarian cancer [48]. circPLEKHM3 binds directly to miR-9 suppressing its activity. By sponging miR-9, circPLEKHM3 also suppresses proliferation and migration of ovarian cancer cells through the upregulation of *KLF4*, which works as a tumor suppressor [48]. 

Competing endogenous RNAs (ceRNAs) define ncRNA molecules interacting with each other in a network that mediates transcriptional regulation and expression levels. ceRNAs are involved in the sponge effect existing between RNA molecules and encompass miRNAs and long non-coding RNAs [49]. Recently, circRNAs have also been involved in the mechanism of sponging and are classified as ceRNAs because of the presence of miRNA response elements (MREs) in their sequences [50]. MREs deplete specific miRNAs by complementarity binding, inhibiting the binding to the 3′-UTR of their target mRNAs. Thus, circRNAs may sequester miRNAs, leading eventually to the suppression of translation repression and mRNA decay [50]. MREs are highly conserved and considered a pan-species regulatory code, pointing to a high relevance of the ceRNA-mediated depletion of miRNAs among the regulatory mechanisms [51]. In this scenario, MREs located in 3′-UTR of coding mRNAs, in their pseudogenes, and within circRNA sequences function as miRNA decoys, antagonizing the repressive activities of the latter [52,53].

**Figure 2:**
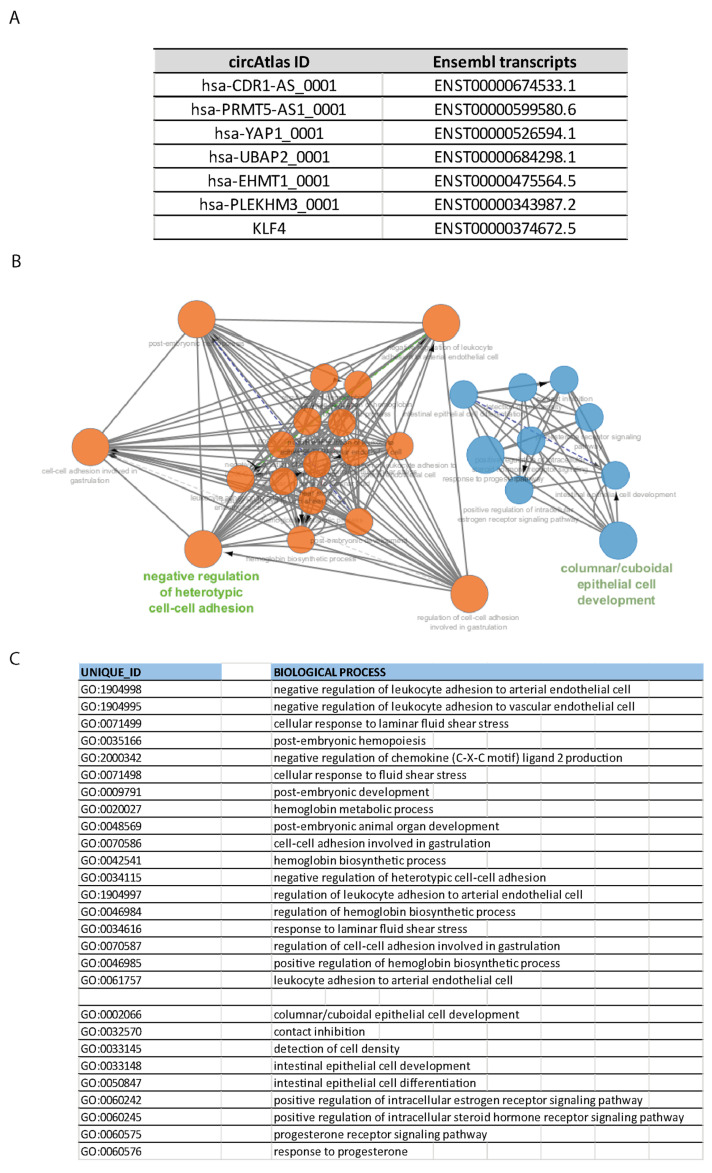
The figure represents the network obtained through the software Cytoscape-ClueGO version 3.10.3. The unique Ensembl transcript IDs of circRNAs and *KLF4* were used to query GO biological processes through ClueGO App. (**A**) Table containing the circAtlas IDs of the circRNAs interacting with *KLF4* as described in the text. The Ensembl transcript IDs are reported. (**B**) Cytoscape version 3.10.3 (cytoscape.org; ClueGO app) was used to search for the interactions. The Ensembl transcript IDs of the circRNAs together with *KLF4*’s were used to build a network of biological processes. (**C**) GO IDs and biological processes that emerged via the ClueGO analysis are reported.

A ceRNA network centered on *KLF4* has been hypothesized by using Cytoscape version 3.10.3 (open source software platform; cytoscape.org) [54]. The circRNAs, identified through circAtlas as mentioned above, correspond to unique Ensembl gene codes [30]. The Ensembl transcript IDs of each circRNA described in this manuscript were used as identifiers and analyzed through the Cytoscape App ClueGO (Figure 2A). ClueGO is designed specifically to perform queries in the Gene Ontology (GO) databases in order to obtain networks. The Ensembl transcript IDs then originated a GO network of biological processes involving *KLF4* and the specific circRNAs, as reported in the figure (Figure 2B,C). The GO unique IDs for each biological process are reported (Figure 2C; cellular components were not considered in the present analysis).

**Figure 3:**
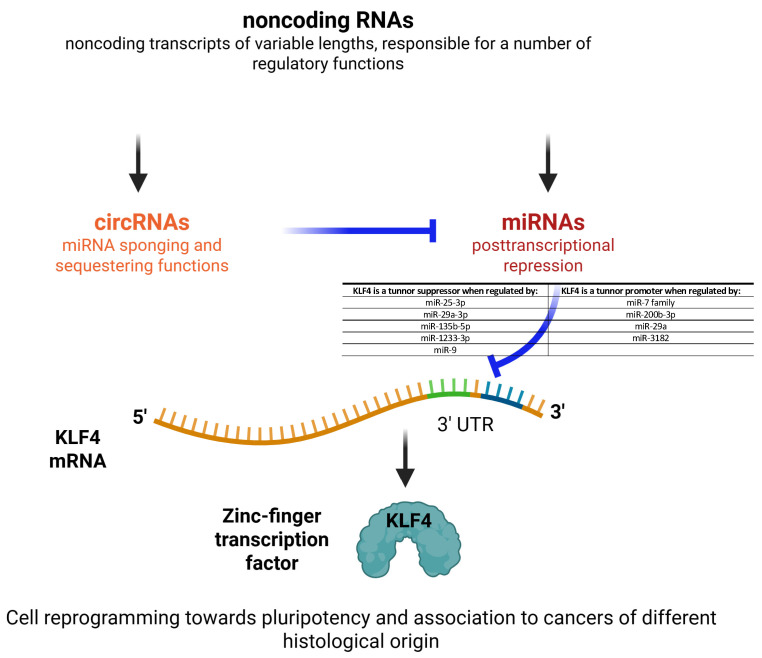
Summary of the interactions occurring among circRNAs, miRNAs, and their target mRNA. These represent a novel layer of epigenetic regulation for *KLF4*. *KLF4* can play either a tumor-promoting or a tumor-suppressive role. Blunt-ends represent inhibitory interactions between the RNA classes indicated in the figure.

A summary representation of the interactions described above is reported in Figure 3. An intriguing hypothesis is that specific circRNA/miRNA axes modulate the tumor-promoting or -suppressive functions of *KLF4* during specific disease stages.

**Table 1:** circRNA/miRNA axes exerting a post-transcriptional regulation of *KLF4* as described in the text. The tumors where the specific functions of the circRNA/miRNA network have been described and the specific references are reported.

circRNA/miRNA	Tumor	References
circ_0071681/miR-29a-3p	Colorectal cancer	[34]
ciRS-7 (CDR1as)/miR-7-5p	Colorectal cancer, esophageal squamous cell carcinoma, hepatocellular carcinoma, hepatoblastoma	[4,31,35,36,37,40]
ciRS-7 (CDR1as)/miR-7	Esophageal carcinoma	[35]
circ_0015756/miR-7	Hepatoblastoma, hepatocellular carcinoma	[41,42,43]
circPRMT5/miR-7-5p	Wilms’ tumor	[20]
circLECRC/miR-135b-5p	Colorectal cancer	[44]
circUBAP2/miR-3182	Non-small cell lung cancer	[46]
circEHMT1/miR-1233-3p	Breast cancer	[47]
circPLEKHM3/miR-9	Ovarian cancer	[48]

## 4. KLF4 May Serve Either as Tumor Promoter or Suppressor During Different Stages of the Disease

The duality of *KLF4*, acting as either a tumor promoter or a tumor suppressor, has been reported by several authors, as summarized above in the present manuscript. This dual role is further complicated by the fact that different stages of a tumor may display different *KLF4* epigenetic states modulating its functions. For instance, *KLF4* expression seems to be higher in normal colon mucosa while it decreases during the adenoma–carcinoma progression of CRC [5,18]. Although preliminary, these data point towards a correlation with CRC prognosis. *KLF4*-positive colorectal cancer patients with lymph node metastasis had a better overall survival than *KLF4*-negative patients with lymph node metastasis [5].

Cancer stem cells (CSCs) are recognized as drivers of CRC development and resistance to chemo- and radiation therapy. CSCs have been reported in the majority of human cancers, and their oncologic role has been demonstrated [18,55,56]. Stemness contributes to therapy resistance and, as is known, *KLF4* expression sustains the CSC phenotype. Among the pathways specifically involved in CSC formation, there is also the Notch pathway, involving the OSKM pluripotency factors; it is responsible for the maintenance of the self-renewal CSC population in BC [55]. Furthermore, the CSC phenotype is repressed by miR-7-5p through an inhibition of stemness-specific proteins (KLF4, CD133, SOX2) [1,18]. miR-7-5p also suppresses proliferation and migration of CRC by targeting *KLF4* [19]. Thus, *KLF4* plays an oncogenic role when downstream of miR-7-5p.

Recent papers have been published supporting the role of *KLF4* in promoting epithelial to mesenchymal transition (EMT) during CRC progression [57]. The KLF4 mechanism of action involves the direct binding and interaction with the *STAT3* promoter, leading to the downregulation of *STAT3* and *p*-*STAT3*. *STAT3* activation, following *KLF4* downregulation, eventually leads to the increase in the EMT markers N-caderin and vimentin in CRC cell lines [57]. According to the manuscript by Yuan L. et al., the downregulation of *KLF4*, through the activation of *p*-*STAT3*, triggers the EMT transition enhancing the proliferative, migratory, and invasive potential of CRC cells [57]. These recent data are therefore consistent with the previous research supporting the tumor-suppressive role of *KLF4* in the adenoma–carcinoma progression of CRC [5,58,59].

The differential and stage-dependent *KLF4* expression was shown several years ago in the cells of the immune system. During the differentiation of Th17 lymphocytes, *KLF4* expressed by differentiated T-lymphocytes undergoes rapid downregulation following activation [60]. Studies supporting this stage-specific expression during differentiation of the B lineage have also been published on B-lymphocytes, B-cell precursors, and plasma cells. *KLF4* is expressed in mature bone marrow plasma cells and, when forced in early plasma cells, induces a specific set of genes associated with plasma cell differentiation [61].

## 5. Concluding Remarks

We go into detail here specifically on the axes circRNA/miRNA/*KLF4* and their implication in tumor development. While several other miRNAs able to regulate linear *KLF4* in different non-neoplastic tissues and cell types have been reported in the scientific literature, those do not fall within the scope of the present manuscript.

One of the most relevant messages emerging in the present manuscript is the fact the circRNAs modulate *KLF4* through the inactivation of the miRNAs positioned upstream in each specific pathway (Table 1; Figure 3). It appears that *KLF4*, when regulated through the axes of the miR-7 family, miR-200b-3p, miR-29a, and miR-3182, plays a role of tumor promoter, favoring and accompanying the proliferation, invasiveness, and therapy resistance of tumors having different histological origins. Conversely, *KLF4* is a tumor suppressor when regulated by miR-25-3p, miR-29a-3p, miR-135b-5p, miR-1233-3p, and miR-9.

This manuscript thus defines the importance of circRNA/miRNA axes in regulating *KLF4* pleiotropic functions in cancer cells at a novel epigenetic level not yet reported thus far. The advent of mRNA- and miRNA-based drugs, together with a better knowledge of the post-transcriptional regulatory mechanisms, offers new and improved opportunities for therapeutic targeting of key molecules. The bioinformatics analysis will be even more indispensable in driving the proper networks during the drug design.

## Abbreviations

***OCT3/4***: Octamer-Binding Transcription Factor 3/4. ***SOX2***: SRY-Box Transcription Factor 2. ***KLF4***: Krüppel-like factor 4. ***c-MYC***: MYC Proto-Oncogene, BHLH Transcription Factor. ***YAP1***: Yes1 Associated Transcriptional Regulator. ***EGFR***: Epidermal growth factor receptor. ***BIRC5***: Baculoviral IAP Repeat Containing 5. ***CTGF***: Connective tissue growth factor. ***NOTCH***: Notch Receptor 1. ***Wnt***: Wingless-Type MMTV Integration Site Family, Member. ***MMP2***: Metalloproteinase-2. ***STAT3***: Signal Transducer and Activator Of Transcription 3. ***p-STAT3***: Phospho-STAT3.
